# *Salmonella enterica* Serovar Dublin from Cattle in California from 1993 to 2019: Characterization and Analysis of Antimicrobial Resistance Diversity

**DOI:** 10.3390/antibiotics13010022

**Published:** 2023-12-25

**Authors:** Richard V. Pereira, Heather M. Fritz, Kathy Toohey-Kurth, Kristin A. Clothier

**Affiliations:** 1 Department of Population Health and Reproduction, School of Veterinary Medicine, University of California, Davis, CA 95616, USA; 2 California Animal Health and Food Safety Laboratory, School of Veterinary Medicine, University of California, Davis, CA 95616, USA; hmfritz@ucdavis.edu (H.M.F.); kaclothier@ucdavis.edu (K.A.C.); 3 California Animal Health and Food Safety Laboratory, School of Veterinary Medicine, University of California, San Bernadino, CA 92411, USA; klkurth@ucdavis.edu

**Keywords:** *Salmonella* Dublin, one health, antibiotic resistance

## Abstract

For this study, antimicrobial susceptibility data for *Salmonella* enterica subsp. enterica serovar Dublin (S. Dublin)—a well-known cattle-adapted pathogen with current concerns for multidrug resistance—were recovered from cattle at the California Animal Health and Food Safety Laboratory System (CAHFS) over the last three decades (1993–2019) and were evaluated using tools to capture diversity in antimicrobial resistance. For this purpose, minimum inhibitory concentration (MIC) testing was conducted for 247 clinical *S*. Dublin isolates. Antimicrobial resistance (AMR) profiles revealed a predominant core multidrug-resistant pattern in the three most common AMR profiles observed. Antimicrobial resistance richness, diversity, and similarity analysis revealed patterns for changes in AMR profiles for different age groups. Discriminant analysis using MIC log2-transformed data revealed changes in MIC for year groups, with a time-sequence pattern observed. Drivers for reduced susceptibility were observed for 3rd generation cephalosporins and quinolones observed for more recent year groups (2011–2015 and 2016–2019) when compared to older year groups (1993–1999 and 2000–2005). Together, these results highlight the changes in the diversity of AMR profiles, as well as changes in susceptibility of *S*. Dublin over time for critical antimicrobials of importance to both animals and humans, and support the need for continued monitoring and efforts that will support judicious use of antimicrobials, especially for these two drug classes.

## 1. Introduction

*Salmonella enterica* serotype Dublin (*S.* Dublin) is a cattle-adapted bacterium that is known for its ability to establish severe infections in both cattle and humans [1,2,3]. The potential risk for food products originating from cattle as a source for *S.* Dublin for humans is a current concern, with recent 2019 outbreaks from ground beef reported by the Center for Disease Control and Prevention (CDC) resulting in multiple human illnesses and deaths [4]. Reports using clinical breakpoints to evaluate changes in antimicrobial resistance (**AMR**) in *S.* Dublin over the years have revealed an increasing incidence of multidrug-resistant isolates [3].

Clinical breakpoints indicate the minimum inhibitory concentration (**MIC**) threshold for categorizing a bacterial isolate as susceptible (S), intermediate (I), or resistant (R). Breakpoints are set for host/drug/organism combinations utilizing integrated knowledge of pharmacokinetic/pharmacodynamic data, distribution of MICs in the population, and the clinical outcome of infections when the antibacterial is used [5]. Due to the complexity, high effort, and cost needed to obtain all of this information, MIC clinical breakpoints have multiple limitations and result in the need for regular updates to maintain their reference values of relevance [6]. These limitations include that veterinary interpretive criteria are generally lacking, particularly for livestock species, and no cattle-specific breakpoints are available for Salmonella. Furthermore, many of the current methods used for monitoring and evaluating resistance have used approaches that focus on the impact or change from resistance to individual antibiotic drugs, rather than the effect and changes occurring for the combination of resistance to multiple antibiotics from which an isolate may be resistant at the same time.

In an effort to evaluate the combined effect of *S.* Dublin isolates for resistance to various combinations of antibiotics, we have utilized methods commonly used for ecological diversity characterization to approach this data. The objectives of this study were to characterize AMR profiles based on clinical breakpoints for *S.* Dublin isolated from cattle in California from 1993 to 2019, evaluating antimicrobial resistance diversity parameters for their composition and distribution. A second objective was to use MIC data for these isolates to identify antibiotics driving the evolutionary distinction over time. We hypothesize that we will identify changes in AMR diversity over time and that specific antibiotics will be identified that differentiate among *S.* Dublin when grouped in year groups.

## 2. Results

### 2.1. AMR Profiles Diversity

The distribution of the antimicrobial resistance profiles of the 247 *Salmonella* Dublin isolates, stratified by year group, is presented in Table 1. The most common AMR profile independent of year group was for the simultaneous resistance to Ampicillin, Streptomycin, Cefoxitin, Amoxicillin/clavulanic acid, Chloramphenicol, Ceftiofur, Ceftriaxone, and Tetracycline (*n* = 42; 17.0%). The second and the third most common MDR profiles had the same aforementioned resistance pattern, with the addition of Gentamicin and Nalidixic Acid, respectively.

### 2.2. Diversity Descriptive Evaluation of AMR Profiles

The calculation of richness and diversity of AMR profiles was applied to describe AMR by response level (Year Group, Age Group, Isolate Source, Clinical Signs, and Region of California) and results are presented in Table 2. The diversity index takes into account both the abundance and evenness of the AMR profiles and can be influenced by sample size. By age group, the largest number of AMR profiles was detected in the early PW group (*n* = 22) and the lowest in the Cow group (*n* = 8). The largest number of AMR profiles by isolate source was observed in feces (*n* = 22) and the lowest number was in the “other” group (*n* = 13). However, the diversity, which accounts for sample size, does not differ appreciably between isolated sources. Diversity was higher among isolates associated with systemic clinical signs compared to those with diarrhea. The diversity of AMR profiles was similar by region of California.

**Figure 1:**
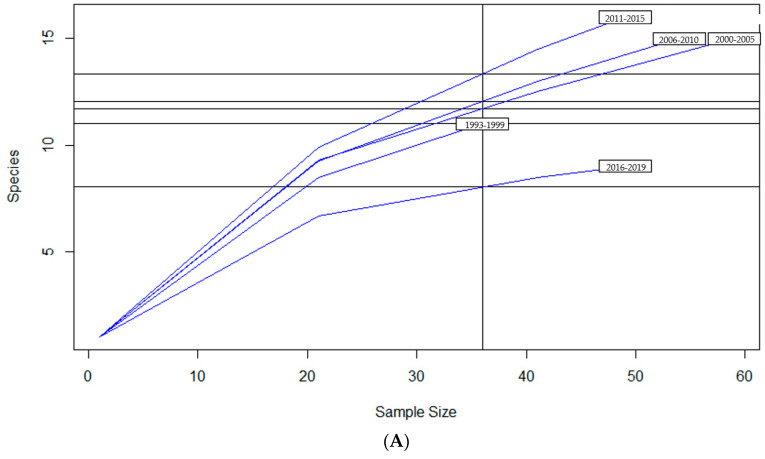

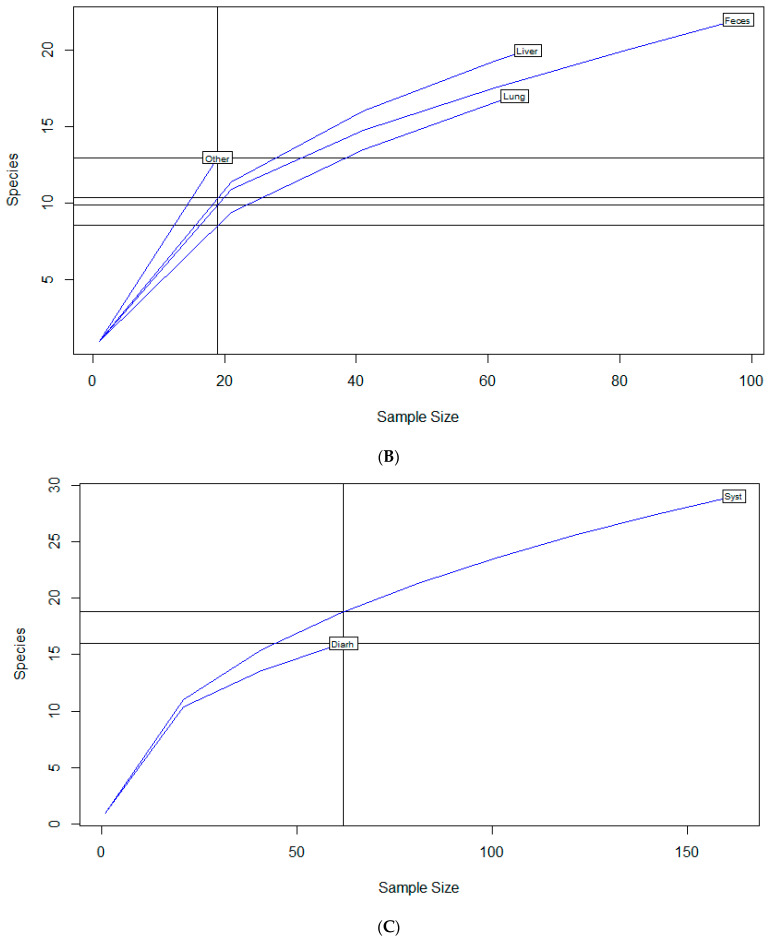

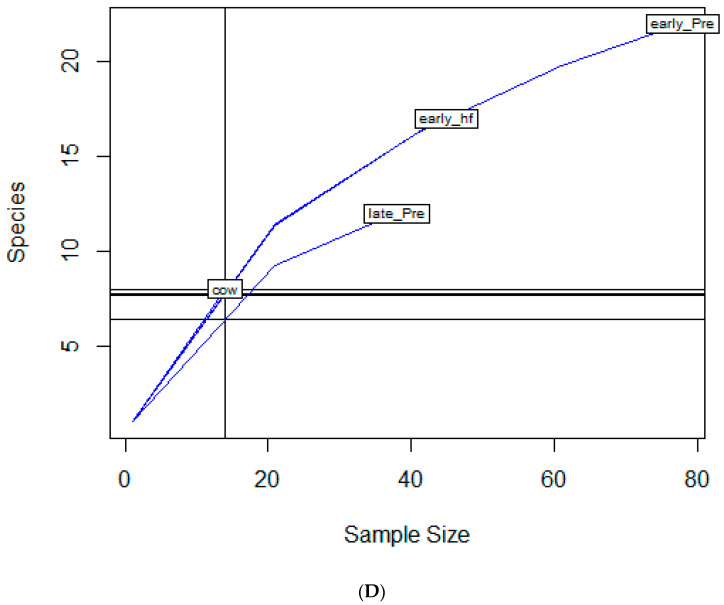
Rarefaction richness curves for *Salmonella* Dublin antimicrobial resistant profile by (**A**) year group, (**B**) isolate source, (**C**) clinical signs, (**D**) age group.

**Figure 2:**
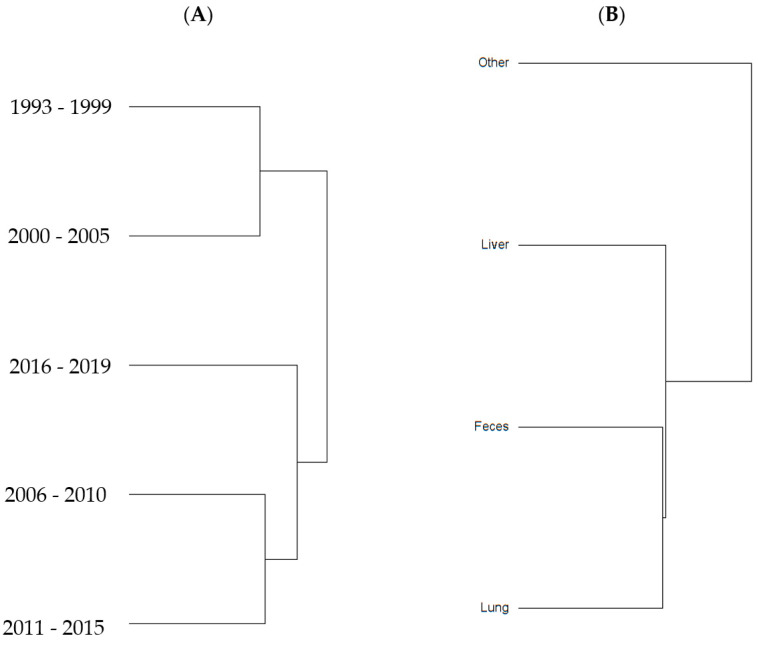
Jaccard similarity clusters for antimicrobial resistant profiles by (**A**) year group and (**B**) source of *S.* Dublin isolates.

**Figure 3:**
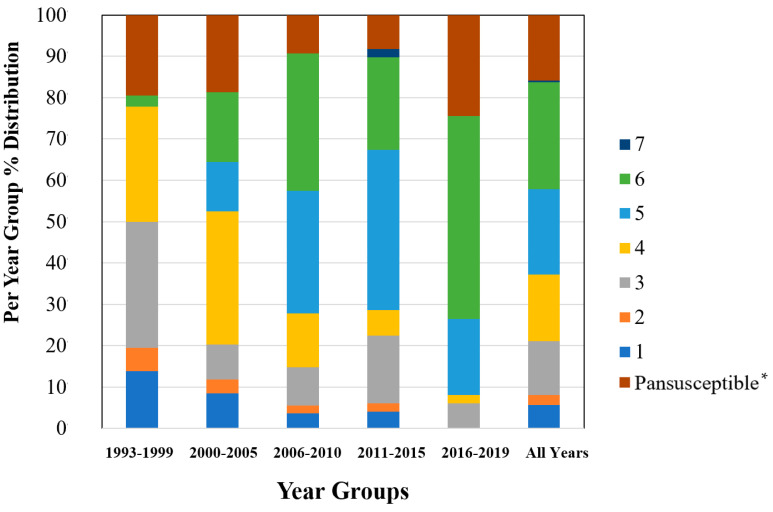
Percent distribution within each year group for the number of antibiotic drug classes for which isolates were resistant for 247 *Salmonella* Dublin isolates using the NARMS gram-negative antimicrobial susceptibility testing panel. * Pansusceptible representing isolates susceptible to all antibiotic drug classes tested.

To examine sample size limitations for AMR profiles for the variables evaluated, rarefaction curves were generated for each response level and illustrate the richness of AMR profiles (each designated as a “species”) at each sample size (Figure 1). The rarefaction curves allow for comparisons between groups of different sample sizes and make it possible to visualize whether the total diversity of AMR profiles was captured in the sample set. Except for the “Other” isolate source and the “Cow” age group, the curves all appear to be plateauing, indicating that species number is not continuing to increase and therefore the sample size was likely sufficient to capture all of the AMR profiles. The evaluation of the Jaccard similarity for AMR profiles is presented in Figure 2, with clustering stratified by year of isolation and isolate source. The percent distribution within each year group for the number of antibiotic drug classes for which isolates were resistant for the 247 *Salmonella* Dublin isolates tested using the United States 1 Antimicrobial Resistance Monitoring System (NARMS) gram-negative panel is presented in Figure 3.

### 2.3. Linear Discriminant Analysis of MIC for Various Factors

**Figure 4:**
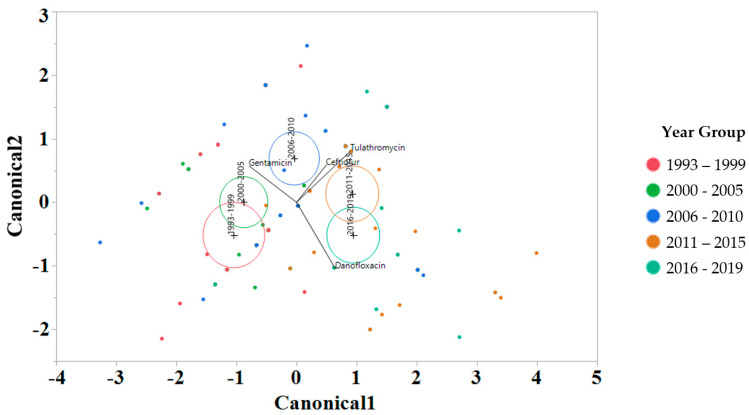
Discriminant analyses of log_2_ minimum inhibitory concentration (MIC) for the antimicrobial drug from the BOPO panel for each year group interval. An ellipse indicates the 95% confidence region to contain the true mean of the group, and a plus symbol indicates the center (centroid) of each group. The length and direction of each biplot ray indicate the degree of association of the corresponding antibiotic with the two canonical variables.

**Figure 5:**
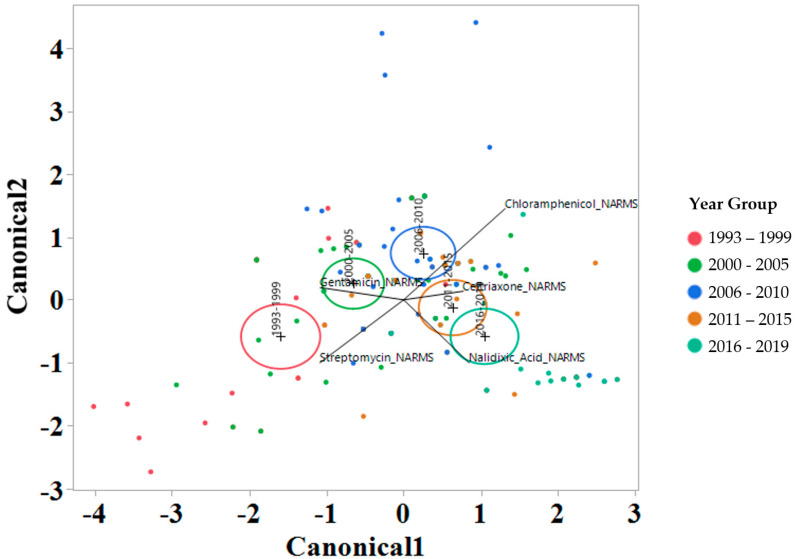
Discriminant analyses of log_2_ minimum inhibitory concentration (MIC) for the antimicrobial drug from the NARMS panel for each year group interval. An ellipse indicates the 95% confidence region to contain the true mean of the group, and a plus symbol indicates the center (centroid) of each group. The length and direction of each biplot ray indicate the degree of association of the corresponding antibiotic with the two canonical variables.

To interrogate which drugs were most influencing the susceptibility trends for each year interval, a linear discriminant analysis (LDA) using log2 MIC data was applied for each antimicrobial drug on the Bovine and Porcine plates (BOPO6F) and NARMS panels (Figure 4 and Figure 5, respectively). This is a novel approach to display the transitions in drug susceptibility of isolates over time and, contrary to traditional methods that relate individual variables to group identity, LDA identifies the combination of variables that best discriminate (distinguish) sample units from different groups. The canonical plot shows the points and multivariate least-square means on the first two canonical variables that best distinguish the groups evaluated. Canonical plots were created for the BOPO6F and NARMS panels, and are shown in in Figure 4 and Figure 5, respectively. Within these plots, biplot rays are present and indicate the directions of the predictors in the canonical space, and each ellipse represents the 95% confidence region to contain the true mean MIC of the group, and the plus symbol indicates the center (centroid) of each group. The length and direction of each biplot ray indicate the directions and degree of association of the predictors in the canonical space, with a longer ray indicating a higher value, while a shorter ray indicates a smaller value. When the circles overlap, they are similar, whereas isolated circles are more significantly different.

## 3. Discussion

This study evaluated antimicrobial resistance diversity in *S.* Dublin based on well-established clinical breakpoints as well as changes in susceptibility to multiple drugs evaluated at the same time using antimicrobial susceptibility data obtained using MIC approach. The most prevalent AMR profiles for isolates evaluated included eight antimicrobial drugs representing six antimicrobial classes and were present in four of the 5 year groups evaluated, with the exception being 1993–1999 when the most prevalent AMR profiles included only three drugs (Table 1). A recent study analyzing AMR profiles for 140 *S.* Dublin isolated from cattle samples submitted to the National Veterinary Services Laboratories (NVSL) from 2014 to 2017 from 21 states observed the same core AMR profile observed in our study [7].

Furthermore, the 2019 Integrated Reports Summary by the NARMS outlined 20 Salmonella isolates that were identified as extremely drug-resistant (XDR) and were defined as isolates resistant to eight or more antimicrobial drug classes [8]. From this NARMS report, 12 of the 20 XDR isolated were *S.* Dublin. An increase in XDR *S* Dublin limits treatment options if of high concern, and longitudinal data have revealed an increased trend toward these multidrug isolates in recent years. In our study, for the year group 2016–2019, the most prevalent AMR profile included the eight AMR drug profiles aforementioned, with the addition of nalidixic acid, increasing the resistance to seven antimicrobial classes with the addition of a drug in the quinolone class. From a risk to human health perspective, the Center for Disease Control and Prevention (CDC) recommends fluoroquinolones, azithromycin, and third-generation cephalosporins as antibiotics of choice for severe salmonellosis, and having highly prevalent *S.* Dublin isolates with resistance to two of those three drug classes highlights the concerns for failure of treatment success [9]. None of the *S.* Dublin isolates in the study demonstrated resistance to azithromycin, and the AMR profile with the broadest impact was resistant to seven drug classes for the 2011–2015 year group (Table 1).

The 2011–2015 year group had the highest number of AMR profiles (*n* = 16), a diversity of 2.2, and an evenness score of 0.8, indicating a fairly even distribution of profiles. By contrast, the number of AMR profiles for 2016–2019 was the lowest of all year groups (*n* = 9), with the lowest diversity (1.7), suggesting that similar AMR profiles are being shared more commonly in *S*. Dublin in more recent years (Table 2). This is a trend that is observed over time, as visualized in the Jaccard similarity for AMR profiles by year group, showing clustering of AMR profiles for 1993–1999 and 2000–2005, 2006–2010, and 2011–2015, with 2016–2019 being similar to the 2006–2010 and 2011–2015 groups but being dissimilar to the point of clustering separately (Figure 2). A concern is that the most common AMR profile for 2016–2019 is an AMR profile with resistance to six drug classes, which includes both 3rd generation cephalosporins and a quinolone drug. The trend for an increase in the percentage of isolates with resistance to five or more drug classes over the years is also reflected in Figure 3, with the vast majority of resistance for isolates from 2016 to 2019 presenting resistance to six antibiotic drug classes.

An interesting finding was a closer similarity in AMR profiles based on Jaccard similarity (Figure 2) between fecal and respiratory samples when compared to hepatic samples. This study was not designed to be able to identify the pathogenesis and route that *S*. Dublin had to be present in the location that it was isolated. However, a hypothesis is that *S*. Dublin from feces and lungs could represent isolates that are more abundant in the environment and would easily have entry to the gastrointestinal tract through the mouth, or to the lung through the mouth or nasal passage. For the case of *S*. Dublin isolated from the liver, the pathogenesis would be from an *S*. Dublin isolate that was able to cause bacteremia and infect the liver tissue, and could represent a sub-type of strain that would not so commonly or not necessarily be identified in the environment, intestine, or lungs. This has been exemplified in calves, where fecal bacterial culture and antimicrobial susceptibility testing are not recommended for calves with diarrhea as fecal bacterial populations do not accurately reflect small intestinal or blood bacterial populations [10].

The stepwise discriminant analysis conducted for MIC data revealed a distinct grouping by year groups, with a clear temporal effect (Figure 4 and Figure 5). This was observed for findings from both BOPO6F and NARMS panels, with slightly different effects, as observed with an overlap of ellipses for 1993–1999 and 2000–2005 in BOPO6F, which was not observed in NARMS, indicating a significant difference. Without the boundaries of clinical breakpoints, these results support a shifting of antibiotic susceptibility indicating an evolutionary trend toward a more common susceptibility pattern, which is observed with isolates from year groups 2011–2015 and 2016–2019 sharing antibiotics. The drivers for the distribution of year groups reveal similar drug classes. For the BOPO panel, four antibiotics are driving the trends over the years tested: gentamicin for 1993–2005, gentamicin and ceftiofur for 2006–2010, ceftiofur and tulathromycin for 2011–2015, and ceftiofur, tulathromycin, and danofloxacin for 2016–2019. On the NARMS panel, streptomycin (not present in the BOPO6F panel) and gentamicin are driving trends for higher MIC in the earlier year groups (1993–1999 and 2000–2005, respectively) and ceftriaxone and nalidixic acid represent the most influential drivers for the distinction of 2011–2015 and 2016–2019 from prior year groups. The approach for simultaneously evaluating the contribution of multiple antibiotics at the same time to identify those leading the evolutionary distinction of *S.* Dublin isolates over the years highlights the need for continued efforts to address factors that have resulted in the reduced susceptibility to critically important antimicrobial drugs. These findings also align with trends observed when using clinical breakpoints for *S.* Dublin for this same dataset, where isolate resistance to third-generation cephalosporins and quinolone drugs have increased over time [11].

## 4. Materials and Methods

### 4.1. Sample Source, Salmonella Identification, and Serotyping

*S.* Dublin isolates were recovered from cattle specimens submitted to the California Animal Health and Food Safety Laboratory (CAHFS) between January 1993 and December 2019. Isolates were obtained from clinically ill animals either at the time of necropsy or from diarrhea samples. Details for the selection process and inclusion criteria, and isolate serotyping using matrix-assisted laser desorption–ionization mass spectrometry (MALDI-TOF; Bruker Daltonics, Fremont, CA, USA) and serovar were confirmed using the Luminex nucleic acid bead-based suspension array and xMAP^®^ Salmonella serotyping assay (Luminex; Austin, TX, USA), outlined in a previous publication [11].

### 4.2. Antimicrobial Susceptibility Testing

The MIC determination was conducted following criteria provided in Clinical and Laboratory Standards Institute (CLSI) documents [12,13] using the Bovine/Porcine (BOPO6F), which includes antimicrobials that are approved for use in food-producing animals, and the NARMS gram-negative panels containing antimicrobials critical to human health (Sensititre, Thermo Fisher Scientific, Waltham, MA, USA), as previously described [11]. The 96 MIC Well Plates were incubated overnight at 37 ± 2 °C. All 247 *S.* Dublin isolates were submitted to these assays. Briefly, clinical antimicrobial resistance interpretation was defined using the Veterinary CLSI-defined (when available for BOPO6F panel drugs) or NARMS consensus breakpoints [12,13]. Drugs tested on the BOP6F panel include ceftiofur (0.25–8 µg/mL), tiamulin (0.5–32 µg/mL), chlortetracycline (0.5–8 µg/mL), gentamicin (1–16 µg/mL), florfenicol (0.25–8 µg/mL), oxytetracycline (0.5–8 µg/mL), penicillin (0.12–8 µg/mL), ampicillin (0.25–16 µg/mL), danofloxacin (0.12–1 µg/mL), sulphadimethoxine (256 µg/mL), neomycin (4–32 µg/mL), trimethoprim/sulfamethoxazole (2/38 µg/mL), spectinomycin (8–64 µg/mL), tylosin (0.5–4 µg/mL), tulathromycin (1–64 µg/mL), tilmicosin (4–64 µg/mL), clindamycin (0.25–16 µg/mL), and enrofloxacin (0.12–2 µg/mL) (only one concentration dilution is used for MIC determination for sulphadimethoxine (256 µg/mL), and methoxazole (2/38 µg/mL)). Drugs tested on the NARMS panel include cefoxitin (0.5–32 µg/mL), azithromycin (0.125–16 µg/mL), chloramphenicol (2–32 µg/mL), tetracycline (4–32 µg/mL), ceftriaxone (0.25–64 µg/mL), amoxicillin/clavulanic acid (1/0.5–32/16 µg/mL), ciprofloxacin (0.015–4 µg/mL), gentamicin (0.25–16 µg/mL), nalidixic acid (0.5–32 µg/mL), ceftiofur (0.12–8 µg/mL), sulfisoxazole (16–256 µg/mL), trimethoprim/sulfamethoxazole (0.12/2.38–4/76 µg/mL), ampicillin (1–32 µg/mL), and streptomycin (2–64 µg/mL).

### 4.3. Statistical Analysis

For analyses conducted, clindamycin, penicillin, sulphadimethoxine, tiamulin, tilmicosin, trimethoprim sulfamethoxazole, and tylosin in the BOPO6F panel were not included because their MIC distribution had more than 95% of isolates within one dilution or the MIC distributions were within fewer than 3 dilutions. Additionally, for the BOPO6F panel, only 2 drugs could be classified using the SIR systems, either because the MIC breakpoints were not contained within the range of concentrations tested or because cattle-specific clinical breakpoints for the drug and organism combination were lacking. The two drugs in the BOPO6F panel for which SIR classification was conducted were ampicillin and ceftiofur.

Isolates were classified into various factors of relevance, including year group (1993–1999; 2000–2005; 2006–2010; 2011–2015; 2016–2019), age group (early pre-weaned (PW); late PW; early heifer (HF); late HF; and adult cow), geographic region (north, central, south), clinical signs (diarrhea vs. systemic illness), and season (winter, spring, summer, and fall). Descriptive data for this distribution have been previously published [11]. For the age group classification, the details for the 6 different categories used are as follows: early pre-weaned (Early PW) when ≤than 4 weeks of age; late pre-weaned (Late PW) when >4 weeks of age and ≤9 weeks of age; early heifer (Early HF) when >9 weeks of age and ≤12 months of age; and adult cattle (Cow) when >17 months of age. The cut-offs selected were based on common dairy cattle management practices that change according to age group, as well as on references used in the National Animal Health Monitoring System (NAHMS) [14]. Another variable created was “Source group”, where isolates isolated from the kidney, spleen, brain, colostrum, milk, ear, and joint fluid were classified as “other”, and the remaining response levels were kept the same, namely feces, liver, lungs, and lymph nodes. This classification was based on the number of samples from the sources of the isolate categorized as “other” having less than 2 isolates originating from that source. For antimicrobials in the NARMS panel, a binomial multidrug resistance variable was also created that defined that if an isolate was resistant to 3 or more different drug classes it was categorized as a “1”, otherwise it is categorized as a “0”. Due to the limited number of CLSI breakpoints available for drugs in the BOPO panel, multidrug-resistant phenotype profiles were not created.

Richness, Shannon’s diversity index, and Jaccard similarity for antibiotic-resistant (AMR) profiles were calculated using Vegan package version 2.5–6 in RStudio (R Foundation for Statistical Computing, Vienna, Austria). AMR profile evenness (Pielou’s J’ evenness) was calculated by dividing Shannon’s diversity index by the natural logarithm of the richness, as previously described [15]. Rarefaction of AMR profile richness was calculated using rarefy in R. AMR profile Jaccard similarity and rarefaction plots were created in R. Value for richness, diversity, and evenness were also stratified by various levels for Year Group, Age Group, Source of Isolate, Clinical Signs and California Region.

For each NARMS and BOPO panel, an analysis of the correlation between serial dilution MIC for each antibiotic and their correlation with year groups was conducted using a linear discriminant analysis (LDA). The two-fold serial dilution MIC data were transformed to base 2 logarithm (log_2_) [16]. Log_2_-transformed MIC data for each antibiotic for each isolate were used as covariates in stepwise discriminant analysis models built in JMP Pro 14.0. Each variable was removed in a stepwise manner until only variables with a *p* value < 0.05 were retained in the final model.

## 5. Conclusions

Together, these results highlight the changes in the diversity of AMR profiles, as well as changes in the susceptibility of *S*. Dublin over time for critical antimicrobials of importance to both animals and humans, namely 3rd generation cephalosporins and fluoroquinolones. Results using Linear Discriminant Analysis showed alignment with trends observed when using clinical breakpoints for *S*. Dublin for this same dataset, where isolate resistance to 3rd generation cephalosporins and quinolone drugs have increased over time, and support the need for continued monitoring and efforts that will support judicious use of antimicrobials, especially for these two drug classes.

## Figures and Tables

**Table 1:** Distribution of antimicrobial resistant profiles of 247 *Salmonella* isolates to the United States National Antimicrobial Resistance Monitoring System (**NARMS**) gram-negative antimicrobial panel, by year group and for all years combined using AMR profile. The heatmap color scale represents a gradual percent distribution (numbers in the cells) by year group, from higher (green) to lower (red) prevalence for a specific AMR profile.

AMR Profile	Nº Drug Classes *	1993–1999	2000–2005	2006–2010	2011–2015	2016–2019	All Years
Count (*n*)		36	59	54	49	49	247
Percent Distribution (by Column)							
PANSUSCEPTIBLE **	0	19.4	18.6	9.3	8.2	24.5	15.8
AmpStrFoxAmcChlXnlCroTet	5	0.0	6.8	24.1	34.7	16.3	17.0
AmpStrFoxAmcChlXnlCroTetGen	5	2.8	15.3	25.9	4.1	0.0	10.5
AmpStrFoxAmcChlXnlCroTetNal	6	0.0	0.0	5.6	10.2	34.7	10.1
AmpStrChlTet	4	13.9	13.6	9.3	0.0	0.0	7.3
AmpStrChlTetGen	4	13.9	18.6	0.0	0.0	0.0	6.5
StrChlTet	3	0.0	1.7	7.4	14.3	6.1	6.1
AmpStrTet	3	25.0	5.1	0.0	0.0	0.0	4.9
Str	1	11.1	8.5	1.9	4.1	0.0	4.9
StrChlNalTet	4	0.0	0.0	0.0	6.1	2.0	1.6
AmpStrAmcChlXnlCroNalTet	6	0.0	0.0	0.0	0.0	8.2	1.6
StrChlTetGen	3	2.8	1.7	1.9	0.0	0.0	1.2
StrFox	2	0.0	1.7	1.9	2.0	0.0	1.2
AmpStrFoxSxtAmcChlXnlCroTet	6	0.0	0.0	0.0	2.0	4.1	1.2
AmpStr	2	2.8	1.7	0.0	0.0	0.0	0.8
AmpStrFoxAmcXnlCroTet	3	2.8	0.0	0.0	2.0	0.0	0.8
AmpStrAmcChlXnlCroTet	5	0.0	1.7	1.9	0.0	0.0	0.8
AmpFoxAmcChlXnlCroTetGen	5	0.0	0.0	3.7	0.0	0.0	0.8
StrFoxSxtChlNalCipTet	6	0.0	0.0	0.0	2.0	2.0	0.8
AmpStrChlNalTetGen	5	0.0	0.0	0.0	2.0	2.0	0.8
StrTet	2	2.8	0.0	0.0	0.0	0.0	0.4
StrGen	1	2.8	0.0	0.0	0.0	0.0	0.4
AmpStrSxtAmcChlXnlCroTetGen	6	0.0	1.7	0.0	0.0	0.0	0.4
AmpStrFoxChlTetGen	5	0.0	1.7	0.0	0.0	0.0	0.4
AmpChlNalTetGen	5	0.0	1.7	0.0	0.0	0.0	0.4
AmpStrSxtAmcChlXnlCroTet	6	0.0	0.0	1.9	0.0	0.0	0.4
AmpFoxAmcXnlCroTetGen	4	0.0	0.0	1.9	0.0	0.0	0.4
AmpFoxAmcChlXnlCroGen	4	0.0	0.0	1.9	0.0	0.0	0.4
Amp	1	0.0	0.0	1.9	0.0	0.0	0.4
AmpStrFoxSxtAmcChlXnlCroNalTet	7	0.0	0.0	0.0	2.0	0.0	0.4
AmpStrFoxAmcChlXnlCroNalTetGen	6	0.0	0.0	0.0	2.0	0.0	0.4
AmpStrFoxAmcChlXnlCroNalCipTet	6	0.0	0.0	0.0	2.0	0.0	0.4
AmpFoxAmcChlXnlCroNalTetGen	6	0.0	0.0	0.0	2.0	0.0	0.4

Amc, amoxicillin/clavulanic acid; Amp, ampicillin; Cro, ceftriaxone; Fox, cefoxitin; Xnl, ceftiofur; Cip, ciprofloxacin; Chl, chloramphenicol; Gen, gentamicin; Nal, nalidixic acid; Str, streptomycin; Tet, tetracycline; Sxt, trimethoprim/sulfamethoxazole. * Number of antibiotic drug classes for which the AMR profile corresponds. ** Isolate susceptible to all NARMS panel antibiotics tested.

**Table 2:** Richness, diversity, and evenness of antimicrobial resistance profiles for *Salmonella* Dublin by year group, age group, source of isolate, clinical signs of the animal when the sample was collected, and region in California where the sample originated.

Variables	Richness ^1^	Diversity ^2^	Evenness ^3^
**Year Group**			
**1993–1999**	11	2	0.85
**2000–2005**	15	2.3	0.84
**2006–2010**	15	2.2	0.81
**2011–2015**	16	2.2	0.8
**2016–2019**	9	1.7	0.79
**Age Group**			
**Early Pre-weaned**	22	2.61	0.84
**Late Pre-weaned**	12	2.19	0.88
**Early Heifer**	17	2.51	0.88
**Cow**	8	1.9	0.91
**Source of Isolate**			
**Feces**	22	2.6	0.84
**Liver**	20	2.6	0.87
**Lung**	17	2.3	0.8
**Other**	13	2.5	0.96
**Clinical Signs**			
**Diarrhea**	16	2.4	0.88
**Systemic**	29	2.7	0.8
**California Region**			
**Central**	23	2.7	0.85
**South or North**	25	2.6	0.8

^1^ Richness: number of different antibiotic resistance profiles for *Salmonella* Dublin. ^2^ Diversity (Shannon’s diversity) index accounts for both the abundance and evenness of the antibiotic resistance profiles for *Salmonella* Dublin. ^3^ Evenness (Pielou’s J’ evenness) for antibiotic resistance profiles for *Salmonella* Dublin.

## Data Availability

This study was funded by the Antimicrobial Use and Stewardship (AUS) Program of the California Department of Food and Agriculture and is subject to California Food and Agricultural Code (FAC) Sections 14400 to 14408. FAC Section 14407 requires that data collected be held confidential to prevent the individual identification of a farm or business; as such, raw data from this study are not able to be shared.
